# Sesamin Ameliorates High-Fat Diet–Induced Dyslipidemia and Kidney Injury by Reducing Oxidative Stress

**DOI:** 10.3390/nu8050276

**Published:** 2016-05-09

**Authors:** Ruijuan Zhang, Yan Yu, Jianjun Deng, Chao Zhang, Jinghua Zhang, Yue Cheng, Xiaoqin Luo, Bei Han, Haixia Yang

**Affiliations:** 1 Department of Nutrition and Food Safety, School of Public Health of Xi’an Jiao Tong University, Xi’an 710061, China; zhangrj@mail.xjtu.edu.cn (R.Z.); yuyan@xjtu.edu.cn (Y.Y.); zhangchao9277@163.com (C.Z.); yyyyyy_214@163.com (J.Z.); chengy@mail.xjtu.edu.cn (Y.C.); miniqiao@126.com (X.L.); hanbei@mail.xjtu.edu.cn (B.H.); 2 Shaanxi Key laboratory of Degradable Biomedical Materials, Department of Food Science and Engineering, College of Chemical Engineering, Northwest University, Xi’an 710069, China; dengjianjun@nwu.edu.cn

**Keywords:** hyperlipidemia, lipid-induced kidney injury, oxidative stress, sesamin

## Abstract

The study explored the protective effect of sesamin against lipid-induced renal injury and hyperlipidemia in a rat model. An animal model of hyperlipidemia was established in Sprague-Dawley rats. Fifty-five adult Sprague-Dawley rats were divided into five groups. The control group was fed a standard diet, while the other four groups were fed a high-fat diet for 5 weeks to induce hyperlipidemia. Three groups received oral sesamin in doses of 40, 80, or 160 mg/(kg·day). Seven weeks later, the blood lipids, renal function, antioxidant enzyme activities, and hyperoxide levels in kidney tissues were measured. The renal pathological changes and expression levels of collagen type IV (Col-IV) and α-smooth muscle actin (α-SMA) were analyzed. The administration of sesamin improved the serum total cholesterol, triglyceride, low-density lipoprotein cholesterol, apolipoprotein-B, oxidized-low-density lipoprotein, and serum creatinine levels in hyperlipidemic rats, while it increased the high-density lipoprotein cholesterol and apolipoprotein-A levels. Sesamin reduced the excretion of 24-h urinary protein and urinary albumin and downregulated α-SMA and Col-IV expression. Moreover, sesamin ameliorated the superoxide dismutase activity and reduced malondialdehyde levels in kidney tissue. Sesamin could mediate lipid metabolism and ameliorate renal injury caused by lipid metabolism disorders in a rat model of hyperlipidemia.

## 1. Introduction

Obesity has become a major global health concern worldwide. It is considered the primary risk factor for developing many chronic disorders such as dyslipidemia, hypertension, and diabetes [1,2]. These conditions may lead to additional complications later, including chronic kidney diseases (CKDs) [3,4]. Meanwhile, CKDs itself is an important contributor to severe cardiovascular damage [5,6,7]. Epidemiological studies revealed that the kidney may be an important link of the interaction between hyperlipidemia and cardiovascular diseases [8,9]. Studies in animal models have shown that lipids modulated the progression of CKDs and may even be important factors in the pathogenesis of renal tissue injury [8,10]. The CKDs are characterized by deposition of lipids in the glomerulus, glomerulosclerosis, epithelial injury, mesangial cell proliferation, accumulation of extracellular matrix, and renal interstitial injury [11]. More recently, a number of potential mechanisms have been proposed to be involved in lipid-induced nephrotoxicity. Lipids may be the primary factor in the pathogenesis of renal tissue injury [8]. Diets rich in cholesterol facilitate the development of kidney injury because cholesterol may accumulate in the kidneys causing oxidative damage—the generation of reactive oxygen species and lipid peroxidation [12]. Antioxidant enzyme activities are inhibited, resulting in antioxidant/oxidant imbalance. Meanwhile, inflammatory stress resulting from oxidative stress may increase cholesterol uptake, inhibit cholesterol efflux, and impair cholesterol synthesis in renal peripheral cells in hyperlipidemic rats [13].

Sesamin, a lignin isolated from sesame seeds and present in sesame oil, has been found to exert beneficial physiological properties [14,15]. The administration of sesamin has been reported to modulate lipid metabolism, reduce cholesterol and oxidation levels [16,17], and protect kidneys in an animal model of renal hypertension with hyperlipidemia [18]. Meanwhile, sesamin increased the activity and mRNA expression of fatty acid oxidation enzymes [19,20,21] and decreased the levels of enzymes involved in fatty acid synthesis in the rat liver [20,21,22,23]. However, the protective effect of sesamin against hyperlipidemia and lipid-induced kidney injury, as well as its underlying mechanisms, remain unknown. This study aimed to explore the protective influence of sesamin in a high-fat diet–induced renal injury rat model, and to clarify the rationale behind the role of sesamin in lipid nephrotoxicity.

## 2. Materials and Methods

### 2.1. Materials

**Figure 1:**
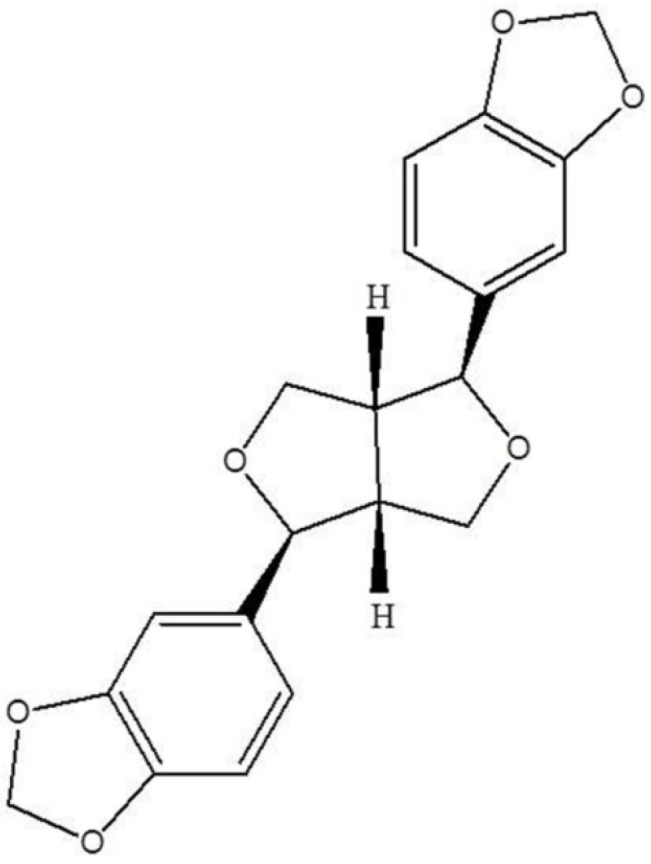
Chemical structure of sesamin.

Sesamin (purity by HPLC > 98.0%) was provided by Shaanxi HuikePhytompharm, Xi’an, China. The structure of sesamin is shown in Figure 1. It was dissolved in plant oil in concentrations of 8, 16, and 32 mg/mL. The stock solutions were prepared once a week and stored at 4 °C. Before gavage, sesamin solutions were acclimated to room temperature, mixed well, and administered to animals in a volume of 5 mL. All chemicals used were purchased from Sigma-Aldrich (St. Louis, MO, USA), unless otherwise indicated. Mouse anti-α-smooth muscle actin (α-SMA) polyclonal antibody and rabbit anti-collagen-IV (Col-IV) polyclonal antibody were all purchased from Boster (Wuhan, China).

### 2.2. Animals

The animal experiment was performed within the jurisdictional framework of the Animal Management Rules of the Ministry of Health of China and the guidelines for the Care and Use of Laboratory Animals of Xi’an Jiaotong University (approval number XJTULAC2012-101; 10 May 2012). Fifty-five adult male Sprague-Dawley rats, 15 weeks of age (160–180 g), were purchased from the Experimental Animal Center of Xi’an Jiaotong University and maintained in separate cages, with five rats per cage. The diet, behavior, and appearance of the animals were recorded daily, and the body weight was measured weekly. Water and food were provided ad libitum, and the cages were maintained at 22 ± 1 °C and 65% relative humidity.

### 2.3. Induction of Hyperlipidemia in the Rat Model

The rats were acclimatized for 1 week before the experiment. The control group (NC) consisting of 11 rats was fed the standard pelleted diet (21% crude protein, 5% lipids, 4% crude fibre, 8% ash, 1% calcium, 0.6% phosphorus, 3.4% glucose, 2% vitamin and 55% carbohydrates). To induce hyperlipidemia, the rats were fed a high-fat diet (73.8% standard diet, 1% cholesterol, 10% yolk powder, 15% lard, 0.2% sodium deoxycholate) for 5 weeks. Subsequently, the rats were randomly divided into four groups, each consisting of 11 animals. One group of rats constituted the hyperlipidemic group (HC) and was fed the standard diet. The other three experimental groups were given a low dose (LDS, 40 mg/(kg·day)), a medium dose (MDS, 80 mg/(kg·day)), and a high dose of sesamin (HDS, 160 mg/(kg·day)) by gavage, once per day, respectively. The rats in the control and hyperlipidemia groups were treated with pure plant oil in parallel. At the end of the 5th and 12th weeks, 24-h rat urine was collected from metabolism cages, and the 24-h urinary protein (24 h-UTP) and urinary albumin (Ualb) were measured using the Protein and Albumin Determination Kit (Jiancheng, Nanjing, China).

### 2.4. Sample Collection

At the end of week 12, the rats were fasted for 12 h and then weighed and anesthetized with an intraperitoneal injection of 30 mg/kg pentobarbital sodium.

The blood was collected through the tail vein after fasting for 12 h, and the serum was isolated. The serum total cholesterol (TC), total triglyceride (TG), high-density lipoprotein cholesterol (HDL-C), low-density lipoprotein cholesterol (LDL-C), apolipoprotein A (apo A), apolipoprotein B (apo B), blood urea nitrogen (BUN), and serum creatinine (SCr) levels were determined using colorimetric enzyme kits (Sigma-Aldrich). Ox-LDL levels were determined using Elisa Kit (Bangyi, Shanghai, China) according to the manufacturer’s protocols in an automatic biochemical analyzer (7170A, Hitachi, Tokyo, Japan).

The left kidney was removed and rinsed with saline. Then, 0.5 cm^3^ of tissue from the middle part of the kidney was excised and fixed in 10% neutral formalin for conventional section by hematoxylin and eosin (HE) and periodic acid—Schiff (PAS) staining. The remaining kidney tissues were rapidly frozen in liquid nitrogen and stored at −70 °C for superoxide dismutase (SOD) and malondialdehyde (MDA) assays. The tissues were then thawed at room temperature and cut into pieces. Next, a prechilled saline solution was added in ninefold excess to prepare the 10% tissue homogenate, which was then further diluted to 1% by prechilled saline.

The SOD and MDA were measured by colorimetric enzyme kits according to the manufacturer’s protocols (Jiancheng, Nanjing, China). The SOD activity was defined as the amount of enzymatic reaction in 1 mL of serum per minute.

### 2.5. Immunohistochemical Analysis of α-SMA and Col-IV Protein Expressions

The renal tissue fixed in 4% buffered paraformaldehyde was embedded in paraffin, and 4-μm-thick sections were stained. For immunohistochemical analysis of α-SMA and Col-IV, the sections were deparaffinized, washed with phosphate-buffered saline, and treated with 3% H_2_O_2_ in methanol for 10 min. All sections were then incubated with the anti-α-SMA antibody (Abcam, Cambridge, UK) (1:100) and the anti-Col-IV antibody (Abcam, Cambridge, UK) (1:100). Next, all sections were incubated with the rabbit anti-mouse biotinylated second antibody immunoglobulin for 30 min, followed by the avidin-biotin peroxidase complex (Maixin Bio, Co., Fuzhou, China). After incubation, the sections were stained with diaminobenzidine (Maixin Bio, Co., Fuzhou, China). The areas positive for α-SMA and Col-IV in the renal tissue were measured. Ten glomerular and tubular areas from each sample were measured, and the positive findings were represented by the integral light density.

### 2.6. Histopathological Examination

The renal tissue sections were mounted on glass slides and stained with HE. Renal pathological changes were detected using the Motic Med 6.0 digital medical image analysis system (Motic, Xiamen, China). The glomerulus (G) and mesangial matrix (M) areas were measured, and the M/G ratio was calculated.

### 2.7. Statistical Analysis

Quantitative data were presented as mean ± standard error of mean (SEM). All data were analyzed by one-way analysis of variance followed by Tukey’s *post hoc* test using the SPSS 13.0 statistical software (SPSS Inc., Chicago, IL, USA). Student’s *t* test was used for comparison between the groups. A *p* value less than 0.05 was considered statistically significant. Non-quantitative results were derived from at least three independent experiments.

## 3. Results

### 3.1. Body Weight, Blood Lipids and Apolipoprotein Changes

**Figure 2:**
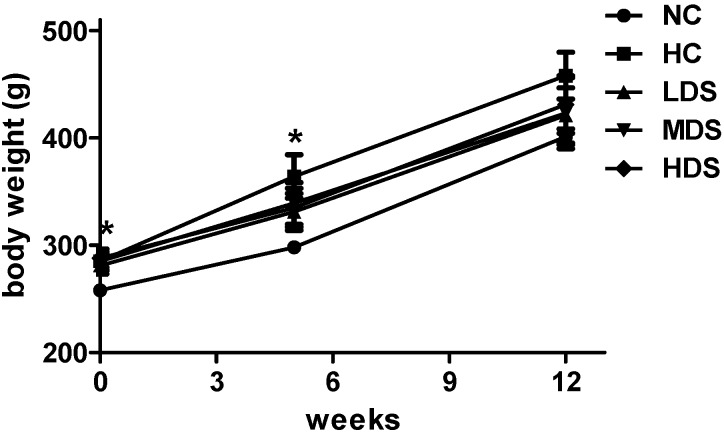
Changes of body weights during the sesamin intervention period. Results are expressed as means ± SEM (*n* = 8–10). * *p* < 0.05 compared with the NC group.

The changes of body weight during sesamin feeding period are shown in Figure 2. The high-fat diet was administered for 5 weeks before the sesamin treatment (0 week in Figure 1). It is shown that the rats belonging to the four groups fed with a high-fat diet significantly increased their body weight compared with the rats in the NC group (*p* < 0.05). However, the body weight of the sesamin treatment groups increased slower than the HC group after 5 weeks and 12 weeks treatment (*p* > 0.05). At the end of sesamin treatment (12 weeks in Figure 2), the TC and TG levels of rats in the HC group (2.62 ± 0.21 mmol/L and 1.22 ± 0.11 mmol/L, respectively) were higher than those in the NC group (1.43 ± 0.12 mmol/L and 0.33 ± 0.14 mmol/L, respectively) (*p* < 0.05), indicating that the dyslipidemia model was successfully established (Table 1). The rats receiving sesamin exhibited lower TC, TG, LDL-C, and Apo B levels and increased HDL-C and Apo A levels (*p* < 0.05) compared with the hyperlipidemia model group. The HDS group exhibited TC levels 36.3% lower (*p* < 0.01), TG levels 23.6% lower (*p* < 0.05), LDL-C levels 20.7% lower (*p* < 0.01), Apo B levels 16.7% lower (*p* < 0.05), HDL-C levels 11.9% higher (*p* < 0.01), and Apo A levels 24.9% lower compared with the HC group (*p* < 0.05) (Table 1). Additionally, high-fat diet significantly increased the serum ox-LDL level compared with NC group (*p* < 0.01). Moreover, ox-LDL levels of MDS and HDS group were reduced in the dyslipidemia model (*p* < 0.05 and *p* < 0.01, respectively).

### 3.2. Renal and Liver Function Indicator Changes

Pathological changes within the kidney tissues were examined, as well as the key indicators of renal function, such as 24 h-UTP and Ualb. After 5 weeks on a high-fat diet, the 24 h-UTP and Ualb excretions increased in the hyperlipidemia group; however, this increase was not statistically significant compared with the NC group (*p* > 0.05). After 12 weeks, the 24 h-UTP and Ualb excretion levels of rats in the HC group significantly increased compared with the control group (*p* < 0.05). The 24 h-UTP excretion in rats from the HDS group significantly decreased compared with the hyperlipidemia model group (*p* < 0.05). The rats in the LDS group also exhibited decreased 24 h-UTP excretion (*p* < 0.05); however, these values did not differ significantly from those in the HC group (*p* > 0.05). In addition, the rats that received all three sesamin doses exhibited reduced Ualb secretion compared with the HC group (*p* < 0.05) (Table 2).

It is evident from Table 3 that both SCr and BUN levels were significantly higher in rats in the HC group than in the NC group (*p* < 0.05) (Table 3). The HDS group had significantly lower SCr levels (*p* < 0.05). Meanwhile, the BUN levels of MDS and HDS groups were lower than those of the HC group (*p* < 0.05). Alanine aminotransferase activity (ALT), as a liver function indicator, were determined after the sesamin treatment. The high-fat diet significantly increased the level of ALT (*p* < 0.01). Sesamin decreased the ALT levels in the three treatment group compared with that in HC group (*p* < 0.05).

### 3.3. Renal Histological Changes

**Figure 3:**
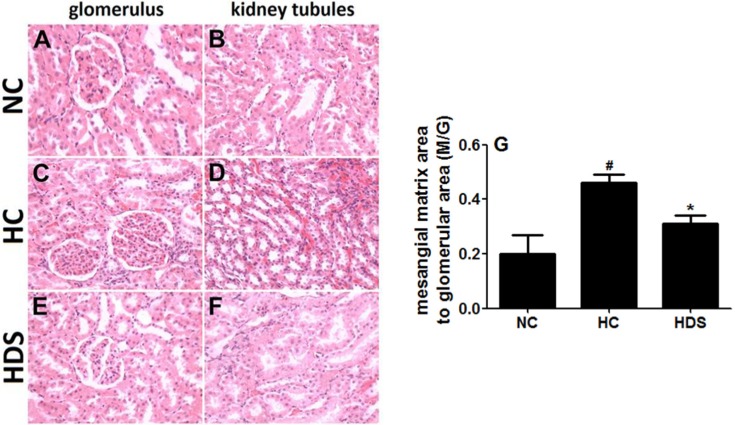
HE staining of renal tissues of rats in different groups. Glomerulus (left) and kidney tubule (right) tissues of rats in the NC group (**A**,**B**) HC group (**C**,**D**) and HDS group (**E**,**F**) (magnification ×400); (**G**) mesangial matrix area to glomerular area (M/G) ratio in different groups. ^#^ *p* < 0.05 compared with the NC group; * *p* < 0.05 compared with the HC group.

The examination of the HE-stained sections of the kidney (Figure 3) in the NC group showed the typical architecture of the kidney glomerulus and tubules, and a lower index of the mesangial matrix (M/G) (Figure 3G). An increased number of glomerular cells in the kidney were observed in the HC group. Also, the glomerular mesangial matrix increased, and obvious epithelial proliferation of kidney tubules was observed (*p* < 0.05). The hyperplasia of mesangial cells and the matrix improved in the three groups when sesamin was administered. The M/G indexes of MDS and HDS groups were significantly lower than those of the hyperlipidemia group (*p* < 0.05).

The SOD activity and MDA levels of rats in the HC group increased significantly compared with the NC group (*p* < 0.05 and *p* < 0.01, respectively). The rats from the HDS group had significantly reduced MDA levels (*p* < 0.05). The SOD activity was higher in the sesamin treatment groups compared with the hyperlipidemia group. However, these differences were not statistically significant (Table 4).

### 3.4. α-SMA and Col-IV Expression in Renal Tissues

**Figure 4:**
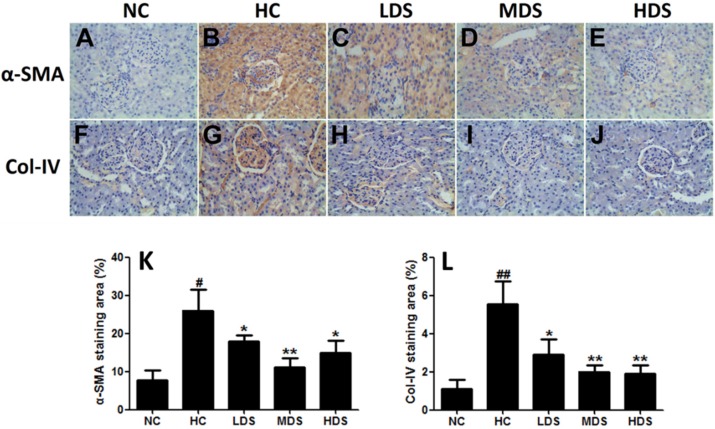
Immunohistochemical staining of α-SMA (**A**–**E**) and Col-IV (**F**,**G**) expression of renal sections in the NC group (**A**,**F**) HC group (**B**,**G**) LDS group (**C**,**H**) MDS group (**D**,**I**) and HDS group (**E**,**J**) (magnification ×400). Quantification of α-SMA (**K**) and Col-IV (**L**) staining, evaluated with Image-Pro Plus software (Media Cybernetics, Silver Springs, MD, USA). ^#^ *p* < 0.05, ^##^ *p* < 0.05 compared with the NC group; * *p* < 0.05, ** *p* < 0.01 compared with the HC group.

Upon additional immunohistochemical staining for α-SMA and Col-IV expression in the renal tissues (Figure 4), low α-SMA and Col-IV expression was observed in rats from the NC group, but high α-SMA and Col-IV expression was detected in the mesangium, tubules, and tubule interstitium of rats from the HC group. Quantification analysis revealed that the α-SMA and Col-IV expression levels were significantly lower in the sesamin treatment groups than in the HC group (*p* < 0.05) (Figure 4K). In addition, the level of Col-IV expression correlated with the dose of sesamin administered (Figure 4L).

## 4. Discussion

This study was the first to show that sesamin improved the lipid profile and led to protection from renal injury due to its antioxidant effect in the rat model with high-fat diet intake.

A rat model correlating obesity-related renal disease and lipid metabolism was successfully established in early studies [2,24,25]. It was characterized by proteinuria, hypertrophy, and increased serum lipid levels. Feeding the rats with a high-fat diet for 5 weeks led to a significant increase in the serum TC and TG levels, indicating hyperlipidemia. The 24 h-UTP and Ualb excretion increased, but not as significantly as previously reported [26]. Also, a continuous high-fat diet for 12 weeks induced a further increase in the serum TC and TG levels, and the increased LDL-C and Apo B levels indicated the stable establishment of hyperlipidemia and a lipid-induced renal injury rat model. Significantly elevated 24 h-UTP and Ualb excretion, as well as serum BUN, SCr, α-SMA, and Col-IV expression in renal tissues, accompanied by pathological changes, further demonstrated that the high-fat diet could induce a classic histological injury in renal structures, including glomeruli, tubules, and the mesangial matrix.

A high-fat diet induces oxidative stress and leads to fatty acid metabolic disorders and renal injury. The administration of sesamin was found to decrease serum TC, TG, LDL-C, and Apo B and increase HDL-C and Apo A levels in a dose-dependent manner in a rat model of hyperlipidemia, suggesting that sesamin plays an important role in regulating lipid metabolism. Ox-LDL promotes the process of some metabolism diseases associated with oxidative stress [27,28]. As we expected, sesamin can protect the LDL against oxidation maybe contributing to the beneficial effect. Meanwhile, the administration of sesamin caused a recovery in the levels of MDA and SOD activity, suggesting a strong antioxidant effect of sesamin in rat renal tissues. Sesamin has been found to modulate lipid metabolism and cause a decrease in cholesterol and oxidation; however, the mechanism by which it does so was not identical. Hirose *et al.* demonstrated that sesamin increases the oxidation rate of fatty acids and enhances the activity and gene expression of fatty acid oxidase in the liver through the peroxisome proliferator-activated receptor-α pathway [29]. In this study, the dosages of sesamin in rats were determined according to some experiments *in vivo* and *in intro* [30,31]. The human dosages (6.4, 12.9, and 25.8 mg/(kg body weight·day)) calculated by the formula for dose translation based on body surface area [32] is similar to the human intake dosage reported by Wu *et al.* [33].

Lipid peroxide exerts a toxic effect on the glomerular basement membrane, damaging its structure and inducing proteinuria. The reduced 24 h-UTP and Ualb excretion in rats that received sesamin, as well as ameliorated BUN and SCr levels in the HDS group, suggests that sesamin protects renal tissues by improving glomerular filtration and ameliorating the retention of SCr and BUN. These effects may be attributed to the anti-oxidative activity of sesamin. In hyperlipidemia, the filtered blood lipids can aggregate on the glomerulus and transform the glomerular mesangial cells from a quiescent phenotype to a proliferative/secretory phenotype, attaining muscle fiber-like characteristics in the process [34]. In addition, tubular epithelial cells may be capable of transforming into other phenotypes under some pathological conditions, such as hyperlipidemia, which may result in renal fibrosis [35,36,37,38]. The expression of α-SMA and the accumulation of Col-IV are the markers of myofibroblasts in the kidneys of rats suffering from hyperlipidemia [39]. The administration of sesamin ameliorated mesangial cell proliferation, decreased the mesangial matrix index, and reduced the proliferation of tubular epithelial cells, as well as interstitial fibrosis of tubules. The expression of α-SMA and Col-IV was significantly reduced in rats treated with sesamin, indicating that sesamin can protect the renal structures and inhibit the phenotypic transformation of mesentery and tubular epithelial cells. This may be because sesamin regulates blood lipids to reduce lipid deposition in renal tissues. Interestingly, sesamin can also improve the liver dysfunction induced by high-fat diet. Its metabolism may have contributed to the reduction of liver lipid deposition by sesamin.

## 5. Conclusions

To sum up, sesamin could mediate lipid metabolism and ameliorate renal injury caused by lipid metabolism disorders in a rat model of hyperlipidemia. However, the specific molecular mechanism by which sesamin induces its effects on hyperlipidemia-induced diseases needs further investigation.

## Figures and Tables

**Table 1:** Blood lipid levels of rats from different groups after sesamin treatment.

Group	TC (mmol/L)	TG (mmol/L)	HDL-C (mmol/L)	LDL-C (mmol/L)	Apo A (g/L)	Apo B (g/L)	Apo A/Apo B	Ox-LDL (μmol/L)
NC	1.43 ± 0.03	0.33 ± 0.04	0.54 ± 0.01	0.92 ± 0.05	0.42 ± 0.01	0.26 ± 0.01	1.60 ± 0.08	4.98 ± 0.03
HC	2.62 ± 0.07 ^##^	1.22 ± 0.04 ^##^	0.40 ± 0.02 ^#^	1.30 ± 0.09 ^##^	0.28 ± 0.02 ^##^	0.44 ± 0.01 ^##^	0.64 ± 0.07 ^##^	7.53 ± 0.12 ^##^
LDS	2.38 ± 0.04	1.05 ± 0.05	0.39 ± 0.01	1.24 ± 0.07	0.31 ± 0.01	0.40 ± 0.02 *	0.77 ± 0.05	7.24 ± 0.28
MDS	1.91 ± 0.05 **	0.96 ± 0.06	0.45 ± 0.01	1.21 ± 0.12	0.33 ± 0.01 *	0.37 ± 0.01 *	0.89 ± 0.05	6.89 ± 0.32 *
HDS	1.63 ± 0.07 **	0.81 ± 0.05 *	0.49 ± 0.01 **	1.05 ± 0.07 **	0.35 ± 0.01 *	0.37 ± 0.01 *	0.95 ± 0.07 *	6.27 ± 0.07 **

TC, Total cholesterol; TG, triglyceride; HDL-C, high-density lipoprotein cholesterol; LDL-C, low-density lipoprotein cholesterol; Apo A, apolipoprotein A; Apo B, apolipoprotein B; Ox-LDL, oxidized-low density lipoprotein. Results re expressed as means ± standard error of the mean (*n* = 8–10). ^#^ *p* < 0.05, ^##^ *p* < 0.01 compared with the NC group; * *p* < 0.05, ** *p* < 0.01 compared with the HC group.

**Table 2:** 24 h-UTP and Ualb levels in rats from different groups after high-fat diet (5 weeks) and sesamin treatment (12 weeks).

Group	24-UTP		Ualb	
	5 Weeks	12 Weeks	5 Weeks	12 Weeks
NC	8.48 ± 1.14	10.50 ± 0.87	6.39 ± 0.82	7.17 ± 0.87
HC	8.76 ± 1.05	14.48 ± 1.22 ^#^	7.89 ± 0.63	12.84 ± 1.30 ^#^
LDS	8.72 ± 1.31	12.92 ± 0.85	7.82 ± 0.59	10.05 ± 1.00 *
MDS	8.77 ± 1.09	12.89 ± 1.10	7.86 ± 0.55	10.11 ± 0.59 *
HDS	8.79 ± 0.95	11.39 ± 0.66 *	7.83 ± 0.70	8.49 ± 0.40 *

24-UTP, 24-h urinary protein; Ualb, urine albumin. Results were expressed as means standard error of the mean (*n* = 8–10). ^#^ *p* < 0.05 compared with the NC group; * *p* < 0.05 compared with the HC group.

**Table 3:** BUN, SCr and ALT levels in rats from different groups after 12 weeks.

Group	SCr (μmol/L)	BUN (mmol/L)	ALT (U/L)
NC	35.33 ± 0.66	6.82 ± 0.38	26.11 ± 4.61
HC	42.13 ± 2.36 ^#^	9.73 ± 0.42 ^#^	43.52 ± 4.72 ^##^
LDS	37.00 ± 1.71	8.85 ± 0.26	29.17 ± 2.53 *
MDS	42.50 ± 3.05	7.63 ± 0.34 *	30.82 ± 3.17 *
HDS	34.56 ± 1.32 *	6.87 ± 0.45 *	29.31 ± 5.41 *

SCr, Serum creatinine; BUN, blood urea nitrogen; ALT, alanine aminotransferase. Results were expressed as means ± standard error of the mean (*n* = 8–10). ^#^ *p* < 0.05 compared with the NC group; * *p* < 0.05 compared with the HC group.3.4. Antioxidant Effect of Sesamin in Renal Tissues.

**Table 4:** Oxidative stress parameter levels in the renal tissue of rats after sesamin treatment.

Group	SOD (U/mg prot)	MDA (nmol/mg prot)
NC	67.26 ± 2.21	3.02 ± 0.03
HC	48.71 ± 2.11 ^##^	4.44 ± 0.38 ^#^
LDS	44.25 ± 4.12	3.30 ± 0.23 *
MDS	59.34 ± 6.07	3.18 ± 0.16 *
HDS	53.83 ± 6.03	2.86 ± 0.36 *

SOD, Superoxide dismutase; MDA, malondialdehyde. Results were expressed as means ± standard error of the mean (*n* = 8–10). ^#^ *p* < 0.05, ^##^ *p* < 0.01 compared with the NC group; * *p* < 0.05 compared with the HC group.

## Abbreviations

The following abbreviations are used in this manuscript:

TC: Total cholesterol
TG: triglyceride
LDL-C: low density lipoprotein cholesterol
apo-B: apolipoprotein B
SCr: serum creatinine
CKDs: Chronic Kidney Diseases
ROS: Reactive Oxygen Species
Col-IV: rabbit anti-collagen-IV
HC: hyperlipidemic group
24 h-UTP: 24 h urinary protein
Ualb: urinary albumin
HDL-C: high-density lipoprotein cholesterol
apo-A: apolipoprotein A
BUN: blood urea nitrogen
HE: Hematoxylin and Eosin
PAS: Periodic Acid-Schiff reaction
SOD: Superoxide Dismutase
MDA: Malondialdehyde
ANOVA: analysis of variance
ALT: alanine aminotransferase activity
